# Potential Roles of n-3 PUFAs during Skeletal Muscle Growth and Regeneration

**DOI:** 10.3390/nu10030309

**Published:** 2018-03-05

**Authors:** Bill Tachtsis, Donny Camera, Orly Lacham-Kaplan

**Affiliations:** Mary MacKillop Institute for Health Research, Exercise and Nutrition Research Program, Australian Catholic University, Melbourne, VIC 3000, Australia; bill.tachtsis@myacu.edu.au (B.T.); donny.camera@acu.edu.au (D.C.)

**Keywords:** omega-3, satellite cells, skeletal muscle, ageing

## Abstract

Omega-3 polyunsaturated fatty acids (n-3 PUFAs), which are commonly found in fish oil supplements, are known to possess anti-inflammatory properties and more recently alter skeletal muscle function. In this review, we discuss novel findings related to how n-3 PUFAs modulate molecular signaling responsible for growth and hypertrophy as well as the activity of muscle stem cells. Muscle stem cells commonly known as satellite cells, are primarily responsible for driving the skeletal muscle repair process to potentially damaging stimuli, such as mechanical stress elicited by exercise contraction. To date, there is a paucity of human investigations related to the effects of n-3 PUFAs on satellite cell content and activity. Based on current in vitro investigations, this review focuses on novel mechanisms linking n-3 PUFA’s to satellite cell activity and how they may improve muscle repair. Understanding the role of n-3 PUFAs during muscle growth and regeneration in association with exercise could lead to the development of novel supplementation strategies that increase muscle mass and strength, therefore possibly reducing the burden of muscle wasting with age.

## 1. Introduction

Skeletal muscle is a highly malleable tissue with the capacity to alter its phenotype in response to exercise and nutrient availability [1]. With increasing age, skeletal muscle becomes less responsive to anabolic stimuli, such as resistance exercise and protein feeding. It is thought that this reduced sensitivity to anabolic stimuli, termed ”anabolic resistance”, is implicated in the etiology of sarcopenia, which is the gradual loss of muscle mass with age [2,3,4,5,6,7]. Other factors that are known to contribute to sarcopenia include reductions in circulating sex hormones [8], physical inactivity [9], low grade inflammation [10,11], impairments to neuromuscular junctions [12], and reduced muscle stem cell (i.e., satellite cell) number and function [13]. Sarcopenia is phenotypically associated with decreased muscle fiber size and shifts in fiber type change from fast to slow, resulting in a decrease in maximal muscle force production [14,15]. These changes are also accompanied by a diminished regenerative capacity of the muscle due to a loss in the number and activity of satellite cells in type II “fast” fibers [14,16,17].

**Figure 1:**
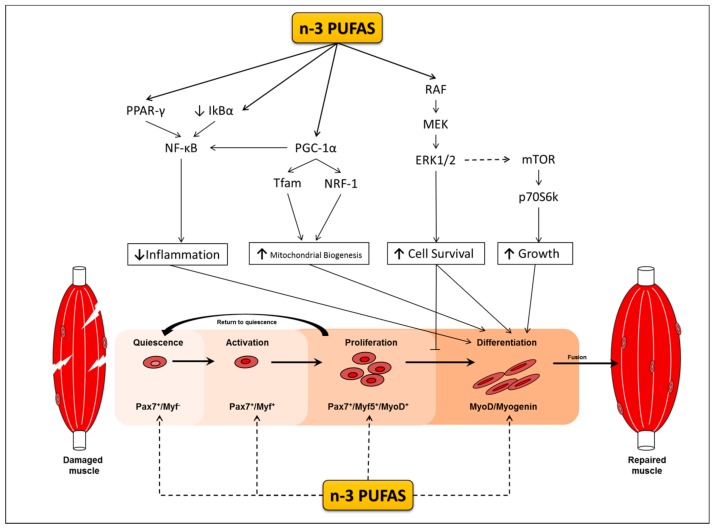
Palmitate (PAL) and Tumour Necrosis Factor-alpha (TNF-α) elicit lipotoxic and cytotoxic deleterious effects on satellite cells during various stages of myogenesis. N-3 PUFAs inhibit the negative effects of PAL and TNF-α by activating anti-inflammatory pathways within the cell thereby promoting differentiation. In isolation, it is currently unknown whether n-3 PUFAs are capable of modulating the expressions of key myogenic transcriptional regulatory factors (MRFs) Pax7, MyoD, and Myogenin that regulate myogenesis. Solid line: represents established role/pathway that n-3 PUFAs modulate during myogenic differentiation. Dotted line: limited or evidence no supporting the role of Omega-3 polyunsaturated fatty acids (n-3 PUFAs) during myogenesis. Solid line: substantial evidence for n-3 PUFAs effecting myogenesis via various pathways.

Satellite cells are muscle specific stem cells primarily responsible for the repair of muscle in response to injury [18,19,20]. Upon their activation, satellite cells enter the cell cycle, proliferate, differentiate to myoblasts and myocytes in a process termed myogenesis, and fuse to damaged muscle fibers. Myogenesis is regulated by changes in the expression of myogenic transcriptional regulatory factors (MRF) that dictate whether satellite cells are in a quiescent, activated, committed, or differentiated state [21,22] (Figure 1). Satellite cells play a role in skeletal muscle repair; however their role in muscle hypertrophy is still equivocal (for reviews see [23,24]). Some animal studies observe skeletal muscle hypertrophy occurring even in the absence of satellite cells [20,25], whereas other animal studies provide evidence to support their role during hypertrophy [26,27]. Although the extent to which satellite cells facilitate muscle hypertrophic response is still a topic of debate, predominating evidence indicates that the presence and more likely the cells’ activation and myogenic capacity are indispensable for supporting training adaptations and may be implicated in the events leading to sarcopenia. However, throughout the lifespan of satellite cell depleted mice, sarcopenia is neither exacerbated nor accelerated [28,29]. Notably, these mice are highly sedentary, and although this may be reflective of an elderly population it is still difficult to draw definitive conclusions in the context of exercise. While satellite cell depleted mice do not show signs of increased muscle loss, satellite cell depletion appears to cause a dysregulation in the surrounding muscle environment leading to increased fibrosis with ageing and a reduction in muscle quality and function. Indeed, satellite cells have been implicated in regulating extracellular matrix production during hypertrophy and regenerative processes, and therefore do play an important role in the maintenance of muscle mass with age [26,30,31]. 

The precise molecular mechanisms responsible for sarcopenia are yet to be elucidated, however, accumulating evidence indicates that nutritional supplements, in particular the omega 3 polyunsaturated fatty acids (n-3 PUFAs), have the potential to reduce muscle wasting and increase the functional capacity of muscle in older individuals by augmenting intracellular anabolic signalling [32,33,34]. Eicosapentaenoic acid (EPA), Docosahexaenoic acid (DHA), and α-linolenic acid (ALA) are the most commonly found n-3 PUFAs in the human diet [35]. ALA is most commonly abundant in plant foods, such as flax seed and nuts, while EPA and DHA are abundant in cold-water fish, such as salmon and tuna [36]. In addition, Docosapentaenoic acid (DPA) is an emerging, but lesser known, n-3 PUFA that possesses a similar structure to EPA and is also biochemically active [37]. Only ALA is considered truly essential to the human diet as both EPA and DHA can be synthesised in the endoplasmic reticulum in liver cells through conversion from ALA, though this conversion is limited due to enzyme availability [38,39,40]. 

The n-3 PUFAs EPA and DHA are well known for their anti-inflammatory properties [41,42]. They have also been reported to elicit positive effects on a wide range of other physiological processes and systems, such as visual signalling [43,44], insulin sensitivity, and glucose tolerance [45]. Accumulating evidence now indicates that n-3 PUFAs may additionally stimulate rates of muscle protein synthesis (MPS) by increasing both the intracellular activities of signalling molecules that are involved in the maintenance of skeletal muscle mass [46,47] and possibly satellite cell activity [48]. Chronic low-grade inflammation associated with ageing and other comorbidities known to increase inflammation, such as obesity, often exacerbate the effects of sarcopenia [49]. As such, the anti-inflammatory effects of n-3 PUFAs on skeletal muscle may be most beneficial to older individuals suffering from sarcopenia [32,50,51] and may complement other strategies, such as exercise and protein supplementation, to combat sarcopenia. In this review, we focus on the dose and treatment periods required for n-3 PUFAs, EPA, and DHA, to support the differentiation of satellite cells into myogenic cells (myogenesis) and elicit protective ant-inflammatory effects in vitro and in vivo. The combined effects of n-3 PUFA supplementation, exercise, and protein ingestion, and their associated mechanisms of action on muscle metabolism in both young and older individuals, will also be discussed.

## 2. The Beneficial Effects of n-3 PUFAs in Skeletal Muscle

### 2.1. Reducing Inflammation

Muscle wasting is a hallmark of various disease states that are often associated with increased levels of inflammation [52,53]. Acute transient increases in inflammation plays an important part in the early regenerative processes of muscle adaptation, particularly in response to exercise [54,55]. However, failure to effectively resolve inflammation at its onset which results in a chronic state of inflammation can be detrimental and is associated with insulin resistance, obesity, and muscle wasting [56,57,58]. Furthermore, elevated levels of inflammation during ageing may also exacerbate the effects of sarcopenia by impairing the regenerative capacity of muscle [59]. 

Upon their incorporation into membrane phospholipids, EPA and DHA act as substrates for the synthesis of lipid derived mediators of inflammation [60]. These mediators, termed eicosanoids, vary in their ability to mediate inflammation depending on the substrate used for their synthesis. For example, eicosanoids derived from arachidonic acid, an omega-6 fatty acid, are considered to be pro-inflammatory [61]. Conversely, eicosanoids derived from n-3 PUFAs are considered to be less potent, reducing the intensity and duration of the inflammatory responses by immune cells [62]. This divergent behaviour is due to the fact that eicosanoids derived from n-3 PUFAs are less biologically active than n-6 PUFAs, and possess a weaker affinity for eicosanoid receptors [63,64,65]. EPA and DHA can also inhibit the endogenous production of pro-inflammatory arachidonic acids by competing as substrates for the enzymes during eicosanoid synthesis [66,67].

Inflammatory cytokines produced by immune cells post-exercise can have a profound effect on skeletal muscle protein turnover and myogenesis. In animal and cell models, cytokines, such as tumour necrosis factor-α (TNF-α) and interleukin-6 (IL-6), bind to receptors on the muscle, activating the transcription factor nuclear factor kappa-light-chain-enhancer of activated B cells (NFκB) [68,69]. Activation of NFκB subsequently increases the expression of the atrogenes muscle RING-finger protein-1 (MuRF1) and atrogin-1, promoting muscle wasting [68]. n-3 PUFAs, in addition to modulating eicosanoid synthesis, can also inhibit the activation of NFκB in skeletal muscle cells and other cell types in vitro (see Section 2.3) [69,70,71,72]. Whether n-3 PUFAs can inhibit the activation of NFκB in human skeletal muscle in response to inflammation remains to be determined. Although being considered pro-inflammatory, TNF-α and IL-6 also appear to play important role in the early stages of muscle regeneration following injury by enhancing myoblast proliferation and inhibiting differentiation [73,74,75]. This inhibition involves the down regulation of the MRFs, MyoD and Myogenin [73,74,75], with TNF-α receptor double knock out mice [74] and with IL-6 non-specific knock out mice [73] shown to present impaired myogenic differentiation and hypertrophy when compared to wild type mice during functional overloading. This highlights the importance of inflammatory processes during muscle regeneration following injury (i.e., exercise). 

On the other hand, chronic inflammation observed during ageing is associated with an increase in pro-inflammatory cytokines (compared to younger individuals) such as TNF-α and IL-6 due to possible dysregulation in immune cell function [59,76]. Elevations in circulating pro-inflammatory cytokines reported in older individuals negatively impacts muscle regeneration by impairing satellite cell differentiation and fusion and by increasing NF-κB activation. These factors contribute to deterioration of muscle mass with age [59,77]. Despite their purported anti-inflammatory effects, which are thought to underlie the beneficial effects of n-3 PUFAs in muscle, Da Boit, et al. [78] and others [33,47], were unable to detect any differences in plasma TNF-α or IL-6 between the placebo and n-3 PUFA treatment groups post-intervention in both older and younger men and women. A possible explanation for this discrepancy is that the participants were healthy and had low levels of inflammation to begin with, making it difficult to detect any changes. 

#### Mechanisms Underlying the Anti-Inflammatory Properties of n-3 PUFAs during Myogenesis

The potential positive effects of n-3 PUFA’s on myogenesis may relate to their capacity to alter the cell membrane lipid composition, which in turn, changes the profile of membrane bound proteins of lipid rafts [79,80]. These changes impact membrane fluidity and assist in myoblast fusion during myotube formation [81], allowing for n-3 PUFAs to simultaneously modulate several signalling pathways. One such pathway involves the Peroxisome proliferator-activated receptors (PPARs), which are a group of nuclear receptors that are well characterised in preventing metabolic disorders, promoting adaptations to skeletal muscle following fasting and physical exercise, and have a novel role in regulating satellite cell activity [82,83,84]. PPARs are also critical regulators of genes that are involved in development, metabolism of lipids and carbohydrates, as well as inflammation [85]. Recent evidence suggests that lipids, such as n-3 PUFAs, are capable of binding to PPARs inducing their activation and altering the expression of pro-inflammatory genes [86]. Results from several studies support the potential for n-3 PUFAs to regulate inflammatory events in muscle cells in association with the PPAR signalling pathway. TNF-α has been shown to elicit negative effects on differentiating myoblasts, resulting in decreases in myotube size and number [87]. However, pre-treating or co-treating myoblasts with EPA prevents the cytotoxic effects of TNF-α [87]. These protective effects of EPA are associated with increases in PPARγ expression and decreases in NF-κB [88]. Moreover, treating C2C12 myotubes with high concentrations (400–600 µM) of EPA or DHA has also been shown to increase the gene expression of PPARγ and reduce muscle breakdown (Huang et al., 2011; Wang et al., 2013). These findings collectively indicate that n-3 PUFAs are able to increase the expression of PPAR and reduce the expression of NF-κB in skeletal muscle resulting in reduced muscle wasting [69,72]. 

PPARδ is another isoform of PPAR abundantly expressed in skeletal muscle that has a novel role in regulating satellite cell activity [89]. In response to injury PPARδ-KO mice when compared to wild type mice, display reductions in proliferating satellite cell number and increases in differentiating cell number [89]. Whether n-3 PUFA supplementation is able to augment satellite cell regeneration in a PPARδ regulated manner following injury or exercise remains to be determined but is likely considering that n-3 PUFAs are known PPARs agonists in skeletal muscle [69].

Like PPARs, the peroxisome proliferator-activated receptor gamma co-activator 1-alpha (PGC-1α) is also capable of repressing the transcriptional activity of NF-kB lowering inflammation in muscle cells [90]. In differentiated C2C12 myotubes, n-3 PUFAs have been found to increase the expression of PGC-1α in a dose and time dependent manner with 50 μM for 24 h having the greatest effect [91]. PGC-1α is also indirectly involved in regulating the expression of mitochondrial DNA (mtDNA) by increasing the transcription of mitochondrial transcription factor A (Tfam) and nuclear respiratory factor 1 (NRF1) [92]. In fully formed myotubes, the expression of both Tfam and NRF1 have been shown to increase following both EPA and DHA treatment [91]. Moreover, overexpressing PGC-1α in C2C12 cells has been shown to promote differentiation and increase the expression of MyoD and Myogenin, however, it remains to be seen whether n-3 PUFAs can elicit similar responses in both PGC-1α and MRF expression during differentiation [93]. 

As fatty acid oxidation occurs within mitochondria, the increases in mitochondrial biogenesis seen following n-3 PUFA treatment could be compensatory as a means of disposing of excess fatty acids more efficiently, which requires more mitochondria [94]. Indeed, EPA and DHA ameliorate the lipotoxic effects of other fatty acids such as palmitate in fully formed myotubes and increase the expression that is associated with mitochondrial-β oxidation, such as carnitine palmitol transferase 1α and β (CPT1α; CPT1β) [95]. Likewise, others have shown that DHA treatment of C2C12 myoblasts attenuates the inhibitory effects of palmitate on PGC-1α activity and preserves oxidative capacity by maintaining citrate synthase activity [96]. Thus, PGC-1α appears to play an important role overcoming the deleterious effects of palmitate lipotoxcity, though it is remains to be determined whether DHA or EPA can modulate the expression of PGC-1α during myoblast differentiation. In Vitro, both PPAR and PGC-1α are capable of attenuating inflammation via downregulating NF-kB following exposure to n-3 PUFAs during myogenic differentiation in C2C12 cells. More work is required to elucidate whether n-3 PUFAs can modulate the same pathways in human satellite cells.

In summary, the n-3 PUFAs EPA and DHA can reduce inflammation via their incorporation into membrane phospholipids, where they inhibit the production of pro-inflammatory eicosanoids, reducing of activation of immune cells and the associated release of pro-inflammatory cytokines [71,97,98]. Therefore, n-3 PUFAs may assist in muscle regeneration in older individuals by reducing excessive inflammation and promoting a systemic environment more conducive to growth [99] (see Section 2.3). The anti-inflammatory properties of n-3 PUFAs and their incorporation into membrane phospholipids may also underpin some of their other beneficial effects in muscle, such as altering protein metabolism, increasing muscle strength and modulating myogenesis. 

### 2.2. Protein Metabolism Regulation

The combination of resistance exercise and protein ingestion is known to maximally stimulate rates of muscle protein synthesis (MPS) [100,101,102] and also lead to increases in satellite cell activity [103] and content in younger men [104]. Moreover, n-3 PUFA supplementation has been shown to exert a positive effect on rates of MPS in both young and old individuals. For instance, when combined with amino acids, n-3 PUFAs enhance rates of MPS in both young and old people to a greater extent than amino acid ingestion alone [33,47]. However, it should be noted that the findings were under non-physiological conditions of a hyperaminoacidemic-hyperinsulinemic clamp [33,47]. Nonetheless, these findings provide evidence that n-3 PUFAs may be an effective strategy to rescue anabolic sensitivity in healthy older individuals purported to be ‘anabolic resistance’, as observed by attenuated MPS responses when compared to healthy young individuals in response to protein ingestion [3,7] and exercise [105]. 

When acute resistance exercise, protein ingestion and n-3 PUFA supplementation (5 g/day for eight weeks) are combined under physiological conditions; rates of MPS are not further enhanced compared to a placebo group in younger adults [46]. Interestingly, the activity of Akt and p70S6K1, signalling kinases involved in translation initiation in muscle, is attenuated following resistance exercise and n-3 PUFA supplementation. This could indicate that anabolic kinase efficiency is increased, suggesting less protein is required to elicit post translational modifications (e.g., phosphorylation) and that these posttranslational modifications occur at a faster rate with n-3 PUFA supplementation. This may be due to the increased incorporation of unsaturated fatty acids into membrane phospholipids thereby changing their composition and altering the activities of proteins that are associated with stimulating MPS tethered to cell membrane [79,80]. 

While it is possible that changes to protein signalling efficiency occur, a major limitation of the study conducted by McGlory et al. [46] is that they did not measure any changes in the total protein content of Akt and p70S6K1 nor did they measure any specific post-translational modifications of these proteins (i.e., altered phosphorylation at numerous sites). Measuring these parameters would confirm whether n-3 PUFAs are indeed capable of altering kinase efficiency and which post-translational modifications are altered following n-3 PUFA supplementation. Lastly, the supplementation protocol (dose and duration) used by McGlory et al. [46] should have been sufficient to induce changes in the muscle. Previously, the same group has shown that after four weeks of supplementation, levels of EPA and DHA continue to rise in muscle (~3.0% of total fatty acids in muscle at week 2 and ~5.0% of total fatty acids in muscle at week 4), whereas levels of EPA and DHA in blood plateau at two weeks with no further increase at four weeks (~8.0% at week 2 and ~8.0% at week 4) [106]. Future research in older individuals is required to investigate whether the combined effects of protein ingestion, resistance exercise, and n-3 PUFA supplementation under physiological conditions is more beneficial than resistance exercise and/or protein ingestion in isolation. It also remains to be seen whether changes in inflammatory markers underlie changes to MPS following n-3 PUFA supplementation.

### 2.3. Increasing Muscle Strength

The central nervous system (CNS) contains a high concentration of the unsaturated lipids, EPA and DHA, which define the structure and function of its cellular and subcellular components. These fatty acids are known to increase nerve conduction velocity in both young and older individuals through the modulation of sarcolemma ion channels which, in turn, improves contractile activity of the muscle [106,107,108,109,110]. In older women, dietary n-3 PUFA supplementation of 2 g/day for 90 days in conjunction with strength training has been shown to exert additional increases to strength and functional capacity (i.e., increased peak torque and rate of torque development) than strength training alone [34]. Rodacki and co-workers [34] also found these increases to occur through changes in neuromuscular junction conductivity, as evidenced by increases in electromyographic activation of the muscle and decreases in electromechanical delay. Even in the absence of regular resistance training, muscular strength and thigh volume can increase with n-3 PUFA supplementation in older men and women. Indeed, supplementation of 4 g/day for six months results in increased whole thigh muscle area, 1-RM strength and isokinetic power [32]. However, in the study that was conducted by Smith et al. diet was not controlled, which may magnify and thus misrepresent the effects of n-3 PUFA supplementation on muscle area [32]. Furthermore, without the collection of muscle biopsies, it is difficult to determine the precise molecular mechanism underlying these responses as well as the potential for similar shifts in muscle fiber types which a known to occur in rat muscle as a result of n-3 PUFA supplementation [111]. 

There is also evidence to suggest that older women may respond better than men to combined n-3 PUFA supplementation and resistance exercise training. For example, when compared to both male and female placebo groups, Da Boit and co-workers observed greater improvement in muscle quality (strength/unit of muscle area) in the quadriceps of older women following combined resistance exercise and fish-oil supplementation [78]. A possible explanation for this sexual dimorphism is that older women do not respond as well to resistance exercise as men and also have less muscle mass, therefore allowing a greater effect in women than men with n-3 PUFA supplementation due to their greater capacity for improvement. This theory however remains highly speculative as others have shown that older men and women respond equally as well to resistance exercise-based training and that no such sex differences exist in older individuals [112].

Furthermore, n-3 PUFAs have been shown to exert similar effects as the female sex hormone oestrogen, preventing bone loss in ovariectomised rats [113]. As such, another possible explanation regarding the sexual dimorphisms in fish oil efficacy is that n-3 PUFAs may function in a similar fashion to oestrogen, promoting anabolism or at least preventing catabolism. However, it should be emphasised here that this study was conducted using an animal model and further human research is required as sexual dimorphisms in protein metabolism are not yet fully understood [114]. Whether n-3 PUFAs induce effects similar to male or female sex hormones in human skeletal muscle related to anabolism remain to be determined. 

### 2.4. Potential to Alter Satellite Cell Activation

Satellite cells are primarily involved in muscle regeneration in response to injury, such as those sustained following resistance exercise [18]. Upon their activation, satellite cells enter the cell cycle, proliferate, and differentiate to myoblasts and myocytes in a process termed myogenesis, before fusing to existing muscle fibers. Circulating systemic factors, such as hormones and inflammatory marker, can positively and negatively influence satellite cell activation. For instance, circulating growth factors, such as GDF11 and myostatin, as well as inflammatory markers, such as TNF-α and IL-6, increase with age, impairing the regenerative capacity of satellite cells in human skeletal muscle [10,115]. Though it is important to note that while GDF11 and myostatin have been correlated with increased ageing in humans, no study to date has demonstrated a causal link between these circulating factors and their ability to affect the regenerative capacity of muscle. Further evidence from parabiosis experiments in mice indicate that ageing reduces satellite cell activity; however exposing cells to a more youthful systemic environment and improves the regenerative capacity of satellite cells indicating that the age-related reduction in satellite cell activity can be modulated by systemic factors that change with age [116,117]. Thus, n-3 PUFAs with their anti-inflammatory properties could promote a systemic environment that improves satellite cell responsiveness to injury during aging by attenuating systemic inflammation in a similar manner to nonsteroidal anti-inflammatory drugs (NSAIDs) [118,119,120]. Currently, there is a paucity of human data to support the latter argument, nevertheless, studies investigating the influence of n-3 PUFAs myogenesis in vitro have been conducted and these findings are summarised in Table 1.

#### The Mechanisms Underlying the Modulatory Effects of n-3 PUFAs on Protein Metabolism Signalling and Myogenesis

Activation of the mechanistic target of rapamycin (mTOR) and its downstream signalling targets the 70 kDa ribosomal protein S6 kinase-1 (p70S6K1) and eIF4E-binding protein 1 (4E-BP1) are involved in the regulation of translation initiation responses that are required for skeletal muscle growth, hypertrophy, and also have a role in myogenesis [121]. In isolation, n-3 PUFAs do not appear to have any effect on mTOR signalling pathway in fully formed C2C12 cells [80]. However, EPA exclusively augments leucine induced MPS in fully differentiated C2C12 myotubes and increases the phosphorylation of p70S6K1, with no changes in the phosphorylation of any of the other anabolic mTOR pathway members [122]. Others have also shown EPA and DHA to induce similar increases in p70S6K1 phosphorylation without leucine stimulation during differentiation [79]. The activation of p70S6K1 following n-3 PUFA treatment in this case is not associated with an increase in Akt activity, which is an upstream effector of mTOR that is implicated in skeletal muscle hypertrophy [79,123]. The fact that changes to p70S6K1 activity [46] and phosphorylation [79] can occur without prior changes to Akt, suggests that n-3 PUFAs are capable of modulating differentiation and MPS independently of canonical mTOR signalling. This alternate activation may possibly come through signalling proteins, such as the mitogen-activated protein kinase (MAPKs), which have been shown to phosphorylate members of the mTOR signalling pathway [124]. 

The MAPK cascade(s), particularly the extracellular signal-regulated kinases (ERK) 1 and 2 (ERK1/2), play a duel role in myogenesis [125,126]. During proliferation, ERK1/2 expression is increased, promoting proliferation and inhibiting differentiation [127,128]. In contrast to this inhibitory effect during the early stages of differentiation ERK1/2 becomes phosphorylated during the late stages of differentiation promoting the fusion and formation of myotubes [129,130]. 

The actions of MAPK signalling in the regulation of myogenesis have been observed in cells treated with n-3 PUFAs. Exposing proliferating C2C12 cells to DHA and EPA for 24 h decreases MAPK/ERK1/2 phosphorylation, preventing the progression of myoblasts from the G_1_ to S phase [131]. When the same cells are proliferated in the absence of n-3 PUFAs for 24 h, they continue through the cell cycle, which is noteworthy as it demonstrates that n-3 PUFAs can regulate a return to quiescence, keeping the cells in the G1 phase. This is noteworthy as there are particular muscle wasting conditions where satellite cells are constitutively active as inflammation is elevated, thus impairing their ability to self-renew [132,133]. Constitutively active satellite cells also cause precocious differentiation (i.e., premature and excessive differentiation), further reducing the satellite cell pool and regenerative capacity of muscle [132,133]. Thus, n-3 PUFAs may have the potential to preserve muscle mass by keeping cells in a quiescent state preserving the satellite pool or at least delay their inevitable activation following the induction of differentiation in vitro. Whether n-3 PUFAs are able to prevent precocious differentiation under pathophysiological conditions in vivo where inflammation is elevated remains to be determined.

Aside from regulating proliferation and differentiation, MAPKs can also initiate cell death responses in the face of noxious stimuli, such as palmitate and TNF-α [134,135]. EPA combined with palmitate and/or TNF-α treatment during C2 differentiation partially rescues cell death and differentiation via the suppression of MAPK [135]. Saini and co-workers [135] also show that EPA promotes differentiation by increasing the expression of MyoD and Myogenin. Whether the other n-3 PUFAs, such as DHA and DPA, elicit the same inhibitory effects on MAPK induced cell death and increase MRFs during differentiation remains unclear. The γ isoform of p38 can also modulate myogenesis by repressing the transcriptional activity of MyoD promoting proliferation [136] although it is unknown whether EPA and DHA alter the activation of p38 MAPK in the context of myogenesis. Future research incorporating in vitro models is required to fully understand the effect that n-3 PUFAs exert on MAPKs during myogenesis and how this affects other pathways that are involved in growth, such as mTOR signalling during this process.

## 3. Limitations and Differences between In Vitro Cell Culture and In Vivo Human Studies

It is evident that EPA and DHA can elicit both similar and differential effects during various stages of myogenesis (Table 1), but also in full differentiated myotubes (Table 2). The differences identified could be due to structural characteristics of the two fatty acids as well as their ability to produce different metabolite species [137,138]. It is important to highlight that the effects of n-3 PUFA in vitro are dose and time dependent and an accurate balance between those parameters is required to significantly augment in vitro proliferation, differentiation, and post-differentiation events. Inconsistency in these parameters may also account for significant differences between published reports. For example, very high concentrations (200–600 μM) of EPA added to mice C2C12 myoblasts during proliferation for up to 10 days induce transdifferentiation to adipocytes [139]. In contrast, EPA has no effect on proliferation rate of C2C12 cells at concentration of 0.1–10 µM even though treated for 48 h, while DHA proliferative stimulation starts at 10 µM [140]. Exposure to EPA and DHA at higher concentrations (20–100 µM) for 24 h slows down the cell growth rate by preventing the transition of C2C12 cells from G_1_ to S phase of the cell cycle with DHA having a more pronounced effect [131]. Additionally, myotubes can be more resilient than myoblasts as concentrations of EPA and DHA, ranging from 400 to 600 µM in myotubes, do not cause protein degradation [69,72] (Table 2). While 50 μM of both EPA and DHA promote myogenic responses during differentiation in C2C12 cells (Table 1), other cell lines such as L6 rat cells are sensitive to 20 μM of EPA and DHA. Furthermore, while studying in vitro myogenesis provides mechanistic insight into the processes underlying muscle development and regeneration, it more closely resembles embryonic muscle development and does not necessarily recapitulate what occurs during myogenesis in adult muscle following injury or exercise. Nevertheless, in vivo human studies investigating the effects on n-3 PUFAs on satellite cell activation are warranted to compliment the in vitro data.

One of the major discrepancies in human studies investigating the effects of n-3 PUFAs alone or combined with exercise and/or protein ingestion is the n-3 PUFA dosage and supplementation duration (Table 3). This discrepancy makes it difficult to elucidate the optimal supplementation duration and the dosage that is required to have an effect on intracellular signalling and muscle anabolism responses. These dose discrepancies may arise due to regional differences in the availability of certain n-3 PUFA supplements, whereas discrepancies in the duration of the supplementation may due to the research question being asked (i.e., acute MPS responses vs chronic changes in lean mass). 

## 4. Future Directions

With respect to muscle fiber hypertrophy, studies concluding that satellite cells are necessary for muscle fiber hypertrophy utilize young or developing mice [146,147] (<4 months of age). However, in models of fully grown/ developed mice (>4 months of age), short-term hypertrophy occurs in the absence of satellite cells [20,24,25]. While satellite cells play an integral role in regulating the extracellular matrix during remodeling [26,30,31], it is yet to be determined whether excessive extracellular matrix (ECM) accumulation or a “myonuclear domain ceiling” limits prolonged hypertrophy in the absence of satellite cells. As such, it is likely that satellite cells are necessary for muscle fiber hypertrophy beyond a certain threshold. With regards to n-3 PUFAs, six months of supplementation in older individuals has been shown to increase gene expression of various pathways that are involved in growth and structural support (ECM organization) [148]. These small n-3 PUFA-induced transcriptional changes in pathways related to growth and ECM organization could modulate satellite cell function given the reciprocal relationship between fibroblasts and satellite cells [31]. Thus, n-3 PUFAs may possess the capacity to alter satellite cell activity directly by altering proliferation and differentiation or indirectly via changes to the ECM. 

Furthermore, the in vitro effects of DPA on myogenesis remain largely understudied. This represents an area for future investigations as DPA is a biologically active n-3 PUFA with similar properties to DHA and EPA and can also be readily converted to EPA [37,149,150]. Thus, further research is required to elucidate whether DPA can also induce myogenesis in a dose dependant manner by regulating the same molecular targets as EPA and DHA. 

Exploring the effects of n-3 PUFAs on human satellite cell myogenesis in vitro as well as ex vivo would provide new insight into the effects timing and dose elicit during the muscle repair process. Understanding precisely how n-3 PUFAs are able to alter anabolic signaling following hypertrophic stimuli, such as protein ingestion and resistance exercise could see novel n-3 PUFA supplementation strategies developed to maximize these responses increasing or at the very least maintaining skeletal muscle mass. 

## 5. Summary and Conclusions

The mechanisms by which n-3 PUFAs exert their multiple beneficial effects center largely on their increased incorporation into cellular membranes which induce changes to various phospholipid species used as substrates for various signalling cascades. n-3 PUFAs are also capable of reducing systemic inflammation by inhibiting the release of pro-inflammatory cytokines from immune cells [151,152,153] and improving the signalling efficiency of proteins that are involved in growth and hypertrophy [46,72,79]. However, several questions remain unanswered regarding n-3 PUFA supplementation and human skeletal muscle metabolism. Firstly, the optimal dosage and duration required to elicit beneficial responses in skeletal muscle remains unclear. As it stands, supplementation dosages ranging from 2 to 5 g/day for a minimum of four weeks results in improvements in anabolic signalling efficiency and muscle strength outcomes (Table 3). In older individuals sex differences in muscle quality have been observed following n-3 PUFA supplementation [78]. The mechanistic basis for these differences remains to be determined. Other sexual dimorphisms may also exist following n-3 PUFA supplementation, such as changes to anabolic signalling activity and rates of MPS, though no study to date has been able to detect these changes. 

Secondly, it is also possible that doses within ranges of 2–5 g/day may affect satellite cell activity particularly after exercise, although experimental evidence is required to confirm this hypothesis. The anti-lipotoxic and anti-cytotoxic properties of EPA and DHA during in vitro myogenesis have been established with 50 µM being an effective concentration to induce protective effects. Whether EPA, DHA, and DPA similarly or differentially modulate the activity and expression of myogenic regulatory factors at 50 µM during myogenesis remains unclear. 

Lastly, the capacity for n-3 PUFAs to activate satellite cells in human skeletal muscle also remains to be determined. This knowledge possesses important health and clinical implications by providing further support for n-3 PUFA supplementation to augment the growth, development and regenerative capabilities of skeletal muscle. Establishing how EPA, DHA, and DPA modulate myogenesis and alters intracellular anabolic signaling would provide a greater understanding as to how n-3 PUFA supplementation can be used as an effective nutritional strategy in conjunction with exercise to increase and maintain muscle mass.

## Figures and Tables

**Table 1:** Summary of studies investigating n-3 PUFAs in proliferating and differentiating myoblasts.

Source	Treatment	Cell Line	Outcome
Smith, et al. [141]	50 μM EPA 1–10 nM PIF	C2C12	EPA ↓ protein degradation in PIF treated myoblasts
Magee, et al. [87]	50 μM EPA 20 ng/mL TNF-α	C2C12	EPA ↓ the negative effects TNF-α via ↓ NF-κB expression. EPA ↑ PPARγ expression
Lee, et al. [140]	1–10 μM DHA 48 h 1–10 μM EPA 48 h	C2C12	DHA ↑ cell proliferation at 10µM concentration only. EPA has no effect on proliferation between 0 and10 µM. EPA and DHA were no investigated in the context of differentiation in this study
Magee, et al. [88]	50 μM EPA 24 h 20 TNF-α ng/mL 24 h	C2C12	EPA ↓ the negative effects TNFα has on cell death, apoptosis and myotube formation by ↑ PPARγ expression via the NfKB signaling pathway.
Peng, et al. [131]	10–100 μM DHA 24 h 10–100 μM EPA 24 h	C2C12	EPA and DHA ↓ cell proliferation in dose dependent manner DHA and EPA ↓ protein levels of Cyclin D and Cyclin E and CDK2. EPA and DHA ↓ ERK1/2 protein expression
Briolay, et al. [79]	20 μM DHA 72 h 20 μM EPA 72 h	L6	Treating cells with EPA and DHA ↑ fusion index. This is accompanied by ↑ in p70S6K1, and membrane bund protein caveolin-3 that is associated with fusion.
Luo, et al. [139]	400–600 μM EPA 10 days	C2C12	EPA in high doses of 400–600 µM induces transdifferentiation of myoblasts to adipocytes by ↓ Wnt/β-catanin signalling through PPARγ
Saini, et al. [135]	50 μM EPA 36 h & 72 h 750 μM PAL 36 h & 72 h	C2	EPA ↓ palmitate induced cell death via affecting the MAPK pathway. EPA can protect cells from MAPK induced cell death and myogenic suppression but not against of Id3/caspase induced apoptosis EPA ↑ MyoD

Abbreviations: Eicosapentaenoic acid (EPA), Docosahexanenoic acid (DHA) Alpha linolenic acid (ALA), Proteolysis inducing factor (PIF), tumour necrosis factor-alpha (TNF- α), nuclear factor kappa-light-chain-enhancer of activated B cells (NF-kB), Peroxisome proliferator activated-receptor gamma (PPARγ), Cyclin dependany kinase 2 (CDK2), Palmitate (PAL). ↑/↓ indicates the trend.

**Table 2:** Summary of studies investigating the effects of PUFAs in full differentiated myotubes.

Publication	Treatment	Outcome
Bryner, et al. [96]	100 μM DHA 500 μM PAL	DHA attenuates ↓ tube size by PAL
Wang, et.al [69]	300–700 μM DHA 300–700 μM EPA	EPA and DHA ↓ total protein degradation in a dose response manner DHA and EPA ↓ protein degradation by regulating the IkBα/NFκB signalling DHA is more effective in preventing degradation than EPA
Kamolrat and Gray [122]	50 μM DHA 50 μM EPA	EPA ↑ MPS and ↓ MPB DHA has no effect on MPS or MPB at 50 μM
Lee, et al. [142]	1–50 μM DHA, 1–50 μM EPA	DHA and EPA both ↑ UCP3 AICAR synergistically ↑ UCP3 activity when co-treated with DHA or EPA
Woodworth-Hobbs, et al. [143]	100 μM DHA 500 μM PAL	DHA ↓ proteolysis by palmitate in a time dependent manner and counteracts the effects of PAL by restoring Akt activity, ↓ FOXO activity ↓ and atrogin expression.
Lee, et al. [91]	1–50 μM DHA 1–50 μM EPA	EPA and DHA ↑ PGC-1α, NRF-1 and Tfam gene expression and ↑ PGC-1α promoter activity
Chen, et al. [144]	50 μM DHA 50 μM EPA, 50 μM AA 750 μM PAL	The negative effects of PAL on AMPK phosphorylation GLUT4 mRNA expression and basal glucose uptake were ↓ by AA, DHA and EPA. The expression of genes associated with protein degradation was ↓ by EPA, DHA and AA
Pinel, et al. [95]	50 μM DHA 50 μM EPA 50 μM ALA 500 μM PAL	EPA and DHA ↑ membrane fluidity which may improve glucose uptake.EPA and DHA ↑ key gene involved in β-oxidation.

Abbreviations: Eicosapentaenoic acid (EPA), Docosahexanenoic acid (DHA) ), tumour necrosis factor-alpha (TNF-α), nuclear factor kappa-light-chain-enhancer of activated B cells (NF-kB), Peroxisome proliferator activated-receptor gamma (PPARγ), Palmitate (PAL), Muscle protein synthesis (MPS) Muscle protein breakdown (MPB), Uncoupling protein-3 (UCP3), 5-Aminoimidazole-4-carboxamide ribonucleotide (AICAR), AMP-dependent protein kinase (AMPK), Forkhead box O (FOXO), Peroxisome proliferator-activated receptor gamma coactivator 1-alpha (PGC-1α). ↑/↓ indicates the trend.

**Table 3:** Summary of human studies that have investigated the effects of n-3 PUFA supplementation combined with resistance exercise and/or protein supplementation on muscle anabolism and muscle function.

Publication	Dose	Duration	Age	Sex	*N*	REX	Protein	Outcomes
Smith, et al. [33]	1.86 g/day EPA 1.5 g/day DHA	8 weeks	39.7 ± 1.7	M	5	-	Insulin clamp Amino Acid clamp	Hyperaminoacidemia-hyperinsulinemia induced MPS ↑ after supplementation and ↑ p-mTOR^Ser2448^ p-p70S6k1^Thr389^.
				F	4			
Smith, et al. [47]	1.86 g/day EPA 1.5 g/day DHA	8 weeks	71 ± 1	M	5	-	Insulin clamp Amino Acid clamp	Hyperaminoacidemia-hyperinsulinemia induced MPS ↑ after supplementation and ↑ p-mTOR^Ser2448^ p-p70S6k1^Thr389^.
				F	3			
Rodacki, et al. [34]	0.4 g/day EPA 0.3 g/day DHA	90 days	64 ± 1.4	F	45	3x/week	-	Fish Oil supplementation and strength training results in ↑ improvements in peak torque and rate of torque development than strength training alone.
		150 days	64 ± 1.4	F	45	3x/week		
McGlory, et al. [106]	3.5 g/ay EPA 0.9 g/day DHA	4 weeks	21.3 ± 3	M	10	-	-	EPA and DHA in the blood ↑ from week 0 to 2 and ↔ from 2 to 4 weeks. Levels of EPA and DHA in skeletal muscle are ↑ from 0 to 2 weeks and further ↑ 2 to 4 weeks.
Smith, et al. [32]	1.86 g/day EPA 1.5 g/day DHA	26 weeks	68 ± 5	M	10	-	-	↑ thigh muscle volume, ↑ hand grip strength and ↑1RM
				F	19			
McGlory, et al. [46]	3.5 g/day EPA 0.9 g/day DHA 0.1 g/day DPA	8 weeks	24 ± 0	M	9	Acute bout	30 g Whey	Compared to placebo no change in myofibrillar MPS following an acute bout of REX and protein ingestion ↓ p70S6K1 and Akt anabolic kinases following REX and protein ingestion.
Da Boit, et al. [78]	2.1 g/day EPA 0.6 g/day DHA	18 weeks	70.6 ± 4.5	M	27	2x/week	-	↑ Maximal isometric torque and ↑ muscle quality in women after exercise training in n-3 PUFA group than in the placebo group, with no such differences in men
			70.7 ± 3.3	F	23			
Lalia, et al. [145]	0.675 g/day EPA 0.3 g/day DHA	16 weeks	27 ± 5	M/F	12	Acute bout	-	Myofibrillar MPS ↑ in both young and old after exercise and supplementation whereas mitoMPS and sarcoMPS ↑ in the older participants
			76 ± 5	M/F	12			

Abbreviations: Eicosapentaenoic acid (EPA), Docosahexanenoic acid (DHA), Docosapentanoeic acid (DPA), Resistance exercise (REX), Muscle protein synthesis (MPS) Muscle protein breakdown (MPB), 1 Repitition Max (1RM),Male (M), Female (F). ↑/↓ indicates the trend.
